# Ewes with higher embryo survival rear lambs that grow faster

**DOI:** 10.1093/tas/txad052

**Published:** 2023-05-15

**Authors:** Paul R Shorten, Anne R O’Connell, Jenny L Juengel

**Affiliations:** AgResearch Limited, Ruakura Research Centre, Private Bag 3123, Hamilton, New Zealand; Headwaters NZ Ltd, Cromwell 9342, New Zealand; AgResearch Limited, Invermay Research Centre, Puddle Alley, Mosgiel, New Zealand

**Keywords:** embryo survival, lamb growth rate, lamb survival, ovulation rate, reproduction, sheep

## Abstract

A key economic driver of a meat producing sheep flock is the total kilograms of lamb liveweight at weaning per ewe exposed to the ram. Optimization of key reproductive steps is required to achieve peak performance of a sheep flock. The goal of this paper was to use more than 56,000 records from a commercial flock to investigate the key reproductive steps affecting flock reproductive performance. We also applied a maximum-likelihood based technique to predict the embryo survival and ovulation rate for daughters of individual sires based on measurements of the number of fetuses at midpregnancy (detected by ultrasound-scanning). The model was used to determine how changes in premating liveweight, age, predicted ovulation rate, embryo survival, number of fetuses at midpregnancy, lamb survival, and lamb growth rate affect the total lamb liveweight at weaning per ewe exposed to the ram in the flock. The data from the commercial flock was also used to investigate the role of ewe age and premating liveweight on each reproductive step. Sensitivity analyses were performed to identify the key reproductive steps affecting flock reproductive performance. The elasticity for embryo survival was 80% of that for lamb survival. There was also significant between sire variance in the estimates of ovulation rate and embryo survival. The reproductive performance of daughters of sires with high (top 50%) and low (bottom 50%) embryo survival was investigated. Embryo survival was 0.88 in the high group and 0.82 in the low group (a 6% reduction in embryo survival). The expected total weight of lambs weaned per ewe exposed to the ram was 42 kg in the high embryo survival group and 37 kg in the low embryo survival group (a 12% reduction in the total weight of lambs weaned per ewe exposed to the ram). The proportion of twin litters was 70% in the high group and 60% in the low group, highlighting the potential importance of embryo survival for the rate of twinning in flocks with ovulation rates greater than two ova. Although lamb survival was similar between the high and low embryo survival groups, lamb growth was reduced by 10% in the low embryo survival group for the same litter size (*P* < 0.001). This novel positive phenotypic association between embryo survival and lamb growth rate can potentially be exploited to improve flock performance.

## INTRODUCTION

The reproductive performance of a sheep flock is an important component of farm production (Young et al., 2014). The reproductive process requires minimal embryo and lamb losses as well as enough lambs and the suitable growth of lambs to weaning. However, there is less information about embryo survival than other reproductive traits as it is a more difficult trait to measure directly. Knowledge of the relationships between ewe age and premating liveweight on the reproductive pathway is available (Oldham et al., 2011; Behrendt et al., 2019), although these relationships are highly variable between research flocks (Hanrahan, 1980; Shorten et al., 2020) and between commercial farms (Shorten et al., 2021). Importantly, ewe age and premating liveweight can be used to predict the reproductive performance of ewes and the survival and growth of their lambs (Cumming, 1977; Hanrahan, 1982). These relationships have been used in models that link environmental, economic, and management variables and the reproduction pathway, which generates important information for an average farm (Amer et al., 1999; Morel and Kenyon, 2006; Young et al., 2011, 2014; Wall et al., 2018), but may not be directly applicable to a specific farm. Improved flock specific characterization of the reproductive process is required for better identification of key targets to improve the reproductive performance of an individual flock (Morel and Kenyon, 2006).

We have previously calculated flock elasticities that describe the relative importance of the effect of average premating ewe liveweight (−0.03 to 0.84), average ovulation rate (0.16 to 0.50), variance in ovulation rate (−0.051 to −0.011), embryo survival (0.51 to 0.77), lamb survival (1.02 to 1.05), conception failure (0.36 to 0.37), and average ewe age (−0.04 to 0.09) on the total kilograms of lamb liveweight at weaning per ewe exposed to the ram in nine commercial flocks (Shorten et al., 2021). Embryo survival had the second largest elasticity, and this highlights that a 1% increase in embryo survival is expected to have a 0.77% increase in the total kilograms of lamb liveweight at weaning per ewe exposed to the ram in one flock. However, there are risks in over extrapolating such results to other farm conditions, and the covariances between embryo survival and other reproductive traits remain largely unknown due to the difficulty of direct measurement of embryo survival. Identification of the covariances between the different reproductive steps will allow for better evaluation of the trade-offs between the reproductive steps for that flock and identification of key targets to improve the reproductive performance of a specific flock and minimize any unintended consequences (Morel and Kenyon, 2006). The heritability of embryo/fetal survival in a research flock based on the direct measurement of ovulation rate and the number of lambs recorded at birth has been established (Shorten et al., 2013), although the widespread commercial use of the embryo survival trait requires easier and more widespread measurement of this trait. The feasibility of a maximum likelihood-based approach to estimate ovulation rate and embryo survival based only on ultrasound information on the number of scanned fetuses at midpregnancy has also been demonstrated (Shorten et al., 2021). This methodology to estimate embryo survival will allow for more widespread breeding for this favorable trait, although the genetic and phenotypic covariances with other important traits such as lamb survival and lamb growth rate are largely unknown and need to be better characterized before the trait is used commercially.

Thus, the first objective of this study was to investigate how ewe age and premating liveweight affect ovulation rate, number of fetuses at midpregnancy, lambing day, lamb survival to weaning, and weaning weight in a New Zealand commercial flock. The second objective was to assess the role of different parameters on the total weight of lambs weaned per ewe exposed to the ram for this flock. The third objective was to investigate the differences in reproductive performance between the daughters of low and high embryo survival sires. Our primary hypothesis is that there are large between sire differences in embryo survival and that the high embryo survival trait is beneficial for the reproductive performance of the flock.

## MATERIALS AND METHODS

### Experimental data

The data consisted of more than 56,000 records from a New Zealand industry flock collected from 2007 to 2020. Records were extracted with permission from the flock owner from the New Zealand Sheep Improvement Limited national performance recording database. Liveweight and body condition score (**BCS**) were measured premating, although liveweight measurements were more abundant than BCS measurements. All ewes were mated under commercial conditions and each year the rams remained with the ewes for approximately two reproductive cycles (34 d). A ram ratio of one ram to around 100 ewes was used in mating groups of 300 to 500 ewes. Ewe lambs were not typically mated, and the analysis was restricted to mature ewes. All ewes were ultrasound-scanned during midpregnancy to determine the number of fetuses. The number of lambs born was not accurately recorded and the number of ultrasound-scanned fetuses was used as a proxy for number of lambs born as losses from scanning to lambing are typically 1% to 2% (Willingham et al., 1986; Shorten et al., 2020). The predicted day of lamb birth was based on fetal size and number measured at ultrasound scanning (Sergeev et al., 1990; Noia et al., 2002). Lamb weaning weight and the predicted lamb age (days) at weaning were also recorded along with the number of lambs weaned per ewe.

Ewes were typically culled for age after five lambing seasons, although low performing ewes (low body condition, udder damage, etc.) were culled at any age to maintain a high-performing flock with a relatively constant size. Nonpregnant ewes were culled and not retained in the flock. Animals were managed under standard commercial conditions and had access to pasture and water to meet their metabolic requirements. Ewes were bred in extensive South Island Hill and High-Country conditions with very limited opportunity for supplementary feeding. The pasture feed varied within and between the years studied, although ewes were managed to remain at an optimal BCS of 3.0 all year round. Ewes were also vaccinated for reproductive diseases.

Rams were sourced from within the flock. Ram selection was primarily based on physical attributes (including sound feet, legs, teeth, meaty rumps, absence of black spots, absence of wool britch, and bareness of head and breech). Rams were a mixture of 1- to 2-yr-old rams (50:50 ratio). Rams were also annually screened for brucellosis and other reproductive faults and diseases.

### Calculation of ovulation rate and embryo survival

Models for calculation of ovulation rate and embryo survival for mature ewes are as described in Shorten et al. (2021). The distribution of ovulation rate can be described by a lognormal distribution $\left(LN(n;\bar{n},\sigma_{n}^{2})\right)$. Embryo survival to lambing can be described by a binomial process (Restall and Griffiths, 1976) with a survival probability that is dependent on the ovulation rate (Geisler et al., 1977). The mean number of ova $\left(\bar{n}\right)$ is typically lower at age 2, increases from age 2 to 5 (Schoenian and Burfening, 1990) and decreases thereafter and can be described by a quadratic function:


$$\begin{array}{l} \bar{n}=\beta_{n0}+\beta_{n1}A+\beta_{n2}A^{2}+\beta_{w1}W+\beta_{w2}W^{2}, \end{array}$$ (1)

with a similar quadratic effect of weight (Morley et al., 1978) (see Table 1 for descriptions and values of parameters). The effect of ewe age on the mean ovulation rate adjusted for premating liveweight is given by equation 1 with $W=\bar{W}$ (where $\bar{W}$ denotes the average liveweight of all ewes). The number of lambs born is therefore described by a compound lognormal–binomial distribution where the probability of observing *k* lambs born is

**Table 1.** Table of model parameters, values, and descriptions for all mature ewes

Parameter	Description, units	Value
β_*n*0_	Intercept term for the relationship between age and weight on ovulation rate, ova	−1.12 ± 0.021
β_*n*1_	Linear effect of age on ovulation rate, ova yr^−1^	0.092 ± 0.017
β_*n*2_	Quadratic effect of age on ovulation rate, ova yr^−2^	−0.0087 ± 0.00223
β_*w*1_	Linear effect of premating liveweight on ovulation rate, ova kg^−1^	0.069 ± 0.00064
β_*w*2_	Quadratic effect of premating liveweight on ovulation rate, ova kg^−2^	−0.00028 ± 0.0000066
σ_*n*_	Standard deviation in ovulation rate, ova	0.48 ± 0.0050
*p*_*n*_	Probability of embryo survival to lambing for single ovulation	0.96 ± 0.0012
µ_W,2_	Mean premating liveweight for 2-yr-old ewe, kg	58.00 ± 0.06
σ_W,2_	Standard deviation in premating liveweight for 2-yr-old ewe, kg	5.79 ± 0.04
µ_W,3_	Mean premating liveweight for 3-yr-old ewe, kg	61.45 ± 0.07
σ_W,3_	Standard deviation in premating liveweight for 3-yr-old ewe, kg	6.08 ± 0.06
µ_W,4_	Mean premating liveweight for 4-yr-old ewe, kg	64.42 ± 0.09
σ_W,4_	Standard deviation in premating liveweight for 4-yr-old ewe, kg	6.67 ± 0.07
µ_W,5_	Mean premating liveweight for 5-yr-old ewe, kg	67.42 ± 0.12
σ_W,5_	Standard deviation in premating liveweight for 5-yr-old ewe, kg	6.29 ± 0.09
µ_W,6_	Mean premating liveweight for 6-yr-old ewe, kg	68.34 ± 0.14
σ_W,6_	Standard deviation in premating liveweight for 6-yr-old ewe, kg	6.44 ± 0.10
µ_W,7_	Mean premating liveweight for 7-yr-old ewe, kg	68.68 ± 0.23
σ_W,7_	Standard deviation in premating liveweight for 7-yr-old ewe, kg	7.00 ± 0.18
µ_W,8_	Mean premating liveweight for 8-yr-old ewe, kg	67.46 ± 0.40
σ_W,8_	Standard deviation in premating liveweight for 8-yr-old ewe, kg	7.03 ± 0.30
µ_W,9_	Mean premating liveweight for 9-yr-old ewe, kg	69.86 ± 0.67
σ_W,9_	Standard deviation in premating liveweight for 9-yr-old ewe, kg	6.51 ± 0.51
*a*_2_	Proportion of ewes in the flock of age 2	0.366
*a*_3_	Proportion of ewes in the flock of age 3	0.248
*a*_4_	Proportion of ewes in the flock of age 4	0.170
*a*_5_	Proportion of ewes in the flock of age 5	0.094
*a*_6_	Proportion of ewes in the flock of age 6	0.076
*a*_7_	Proportion of ewes in the flock of age 7	0.032
*a*_8_	Proportion of ewes in the flock of age 8	0.011
*a*_9_	Proportion of ewes in the flock of age 9	0.003
β_10_	Intercept term for the relationship between age and weight and lamb survival for singles	−1.12 ± 2.41
β_*w*11_	Linear effect of premating liveweight on lamb survival for singles, kg^−1^	0.110 ± 0.080
β_*w*12_	Quadratic effect of premating liveweight on lamb survival for singles, kg^−2^	−0.0010 ± 0.0007
β_*A*11_	Linear effect of age on lamb survival for singles, yr^−1^	0.025 ± 0.22
β_*A*12_	Quadratic effect of age on lamb survival for singles, yr^−2^	0.028 ± 0.030
β_20_	Intercept term for the relationship between age and weight and lamb survival for twins	−0.256 ± 1.06
β_*W*21_	Linear effect of premating liveweight on lamb survival for twins, kg^−1^	0.053 ± 0.034
β_*W*22_	Quadratic effect of premating liveweight on lamb survival for twins, kg^−2^	−0.00045 ± 0.00027
β_*A*21_	Linear effect of age on lamb survival for twins, yr^−1^	0.20 ± 0.077
β_*A*22_	Quadratic effect of age on lamb survival for twins, yr^−2^	−0.00043 ± 0.010
β_30_	Intercept term for the relationship between age and weight and lamb survival for triplets	3.80 ± 1.75
β_*W*31_	Linear effect of premating liveweight on lamb survival for triplets, kg^−1^	−0.078 ± 0.052
β_*W*32_	Quadratic effect of premating liveweight on lamb survival for triplets, kg^−2^	0.00045 ± 0.00038
β_*A*31_	Linear effect of age on lamb survival for triplets, yr^−1^	0.18 ± 0.12
β_*A*32_	Quadratic effect of age on lamb survival for triplets, yr^−2^	0.000043 ± 0.014
β_40_	Intercept term for the relationship between age and weight and lamb survival for quadruplets	70.82 ± 18.7
β_*W*41_	Linear effect of premating liveweight on lamb survival for quadruplets, kg^−1^	−1.72 ± 0.52
β_*W*42_	Quadratic effect of premating liveweight on lamb survival for quadruplets, kg^−2^	0.012 ± 0.0037
β_*A*41_	Linear effect of age on lamb survival for quadruplets, yr^−1^	−4.47 ± 1.46
β_*A*42_	Quadratic effect of age on lamb survival for quadruplets, yr^−2^	0.55 ± 0.175
*G*_*M*_	Growth rate advantage of ram lamb over ewe lamb, kg d^−1^	0.024 ± 0.00073
γ_1_	Lamb growth rate for birth rank 1, kg d^−1^	0.097 ± 0.0094
θ_1_	Effect of premating ewe liveweight on lamb growth rate for birth rank 1, d^−1^	0.0032 ± 0.00016
γ_2_	Lamb growth rate for birth rank 2, kg d^−1^	0.098 ± 0.0040
θ_2_	Effect of premating ewe liveweight on lamb growth rate for birth rank 2, d^−1^	0.0026 ± 0.000064
γ_3_	Lamb growth rate for birth rank 3, kg d^−1^	0.14 ± 0.0097
θ_3_	Effect of premating ewe liveweight on lamb growth rate for birth rank 3, d^−1^	0.0017 ± 0.00014
γ_4_	Lamb growth rate for birth rank 4, kg d^−1^	0.29 ± 0.058
θ_4_	Effect of premating ewe liveweight on lamb growth rate for birth rank 4, d^−1^	−0.00063 ± 0.00082
σ_*L*_	Perturbation amplitude for lamb growth rate, kg d^−1/2^	0.70 ± 0.0031


$$\begin{array}{l} \begin{array}{ll} P\left(k;\bar{n},\sigma_{n}^{2},p_{n}\right)= & \frac{1}{T}\underset{m=1}{\overset{7}{\sum }}\;LN\left(m;\bar{n},\sigma_{n}^{2}\right)\frac{m!}{k!\left(m-k\right)!}{\left(p_{n}-d\left(m-1\right)\right)}^{k}\\ & {\left(1-\left(p_{n}-d\left(m-1\right)\right)\right)}^{m-k},\\ & \;T=\underset{i=1}{\overset{7}{\sum }}\;LN\left(i;\bar{n},\sigma_{n}^{2}\right), \end{array} \end{array}$$ (2)

where *p*_*n*_ is the probability that an embryo survives to lambing when there is initially a single ovum, *d* = 0.1 is the assumed rate of decrease in embryo survival with ovulation rate (Shorten et al., 2013), *T* is a normalization factor, and the maximum ovulation rate is assumed to be seven ova. Embryo survival probabilities were assumed to be in the range 0 to 1. All liveweight effects occur via ovulation rate (equation 1) even though liveweight is known to also affect embryo survival (Shorten et al., 2013). However, liveweight effects on ovulation are significantly greater than liveweight effects on embryo survival (Shorten et al., 2013) and therefore liveweight effects on embryo survival are not directly included in the model.

### Statistical analysis

Ovulation rate (β_*n*0_) and embryo survival (*p*_*n*_) model parameters were estimated for each sire. There was a total of 454 sires and the average number of litters for daughters of sire, each with an associated premating ewe liveweight, was 45 litters. Estimates were only obtained for 208 sires whose daughters had more than 40 litters in total (each litter with an associated premating ewe liveweight measurement). Estimates were based on the distribution of the number of lambs born for mature ewes. Model parameters β_*n*1_, β_*n*2_, β_*w*1_, β_*w*2_ and $\sigma_{n}^{2}$ were estimated for the entire dataset. Ovulation rate (β_*n*0_) and embryo survival (*p*_*n*_) model parameter estimates were therefore adjusted for ewe age at mating and premating liveweight (other covariates and factors could potentially be incorporated in the model estimation procedure if required). Maximum likelihood (Pawitan, 2001) was employed to obtain estimates of ovulation rate and embryo survival for each sire. Model parameters for the effects of premating liveweight and age on lamb survival and lamb growth were also estimated based on the methodology of Shorten et al. (2021). Flock elasticities that describe the relative importance of the effect of average premating ewe liveweight, average ovulation rate, variance in ovulation rate, embryo survival, lamb survival, conception failure, and average ewe age on the total kilograms of lamb liveweight at weaning per ewe exposed to the ram were also calculated (Shorten et al., 2020, 2021). Calculations were conducted in Matlab (The Mathworks).

Between sire variability in ovulation rate (β_*n*0_) and embryo survival (*p*_*n*_) model parameters were determined with a linear mixed model with sire as a random effect, sire birth year as a random effect, and regression weights inversely proportional to the square of the standard error associated with each estimate.

Calculations were conducted with 1) all data; 2) daughters of sires with high (top 50%) embryo survival; and 3) daughters of sires with low (bottom 50%) embryo survival.

## RESULTS AND DISCUSSION

### Number of fetuses at midpregnancy

**Figure 1.**
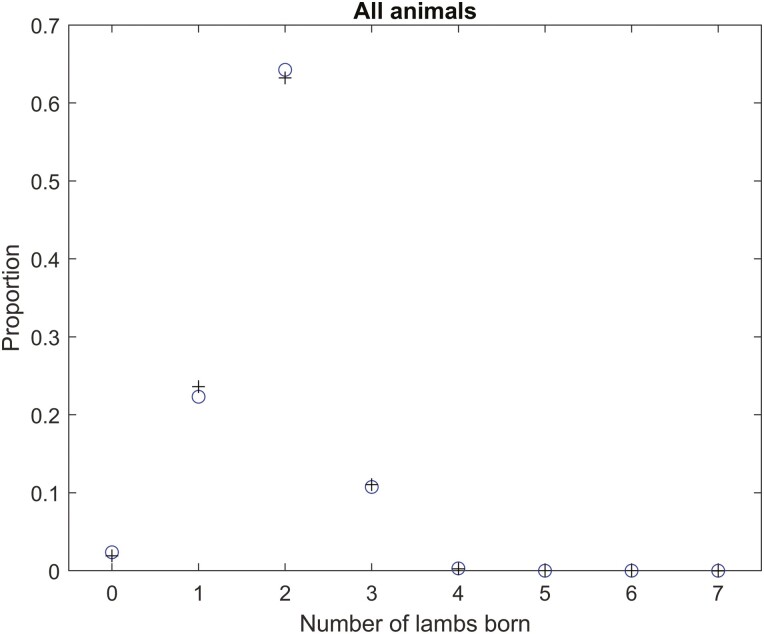
The distribution of the number of lambs born for all mature ewes (circles) and the compound lognormal–binomial model distribution fit (plus symbols) to the data (adjusted for age and weight effects on the number of ova via equations 1 and 2). The mean number of lambs born was 1.84 and the standard deviation in the number of lambs born was 0.65.

The distribution of the number of fetuses at midpregnancy is shown in Fig. 1. The compound lognormal–binomial distribution adjusted for age and weight effects provided a good description of the distribution of the number fetuses at midpregnancy (Fig. 1). The mean number of lambs born was 1.84 and the standard deviation in the number of lambs born was 0.65. The proportion of ewes with twins was 65%.

### Embryo survival and ovulation rate

**Figure 2.**
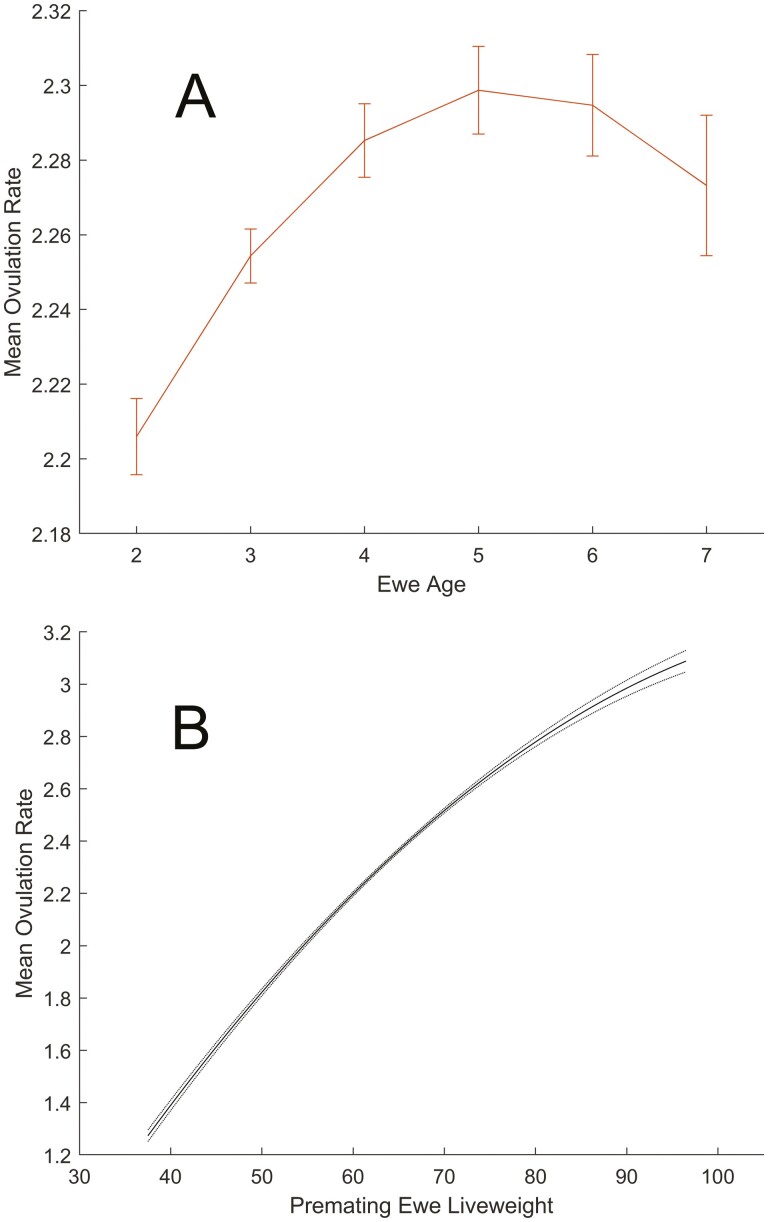
(A) Effect of ewe age on ovulation rate adjusted for premating liveweight, kg for all ewes. Error bars denote SEM. (B) Effect of premating ewe liveweight on ovulation rate adjusted for ewe age for all ewes. Dotted lines denote SEM. Curves are plotted over the range of values observed for all ewes.

There were significant quadratic effects of ewe age and premating liveweight (*P* < 0.001) on ovulation rate (Fig. 2; Table 1) consistent with estimates from other studies (Thompson et al., 1985; Davis et al., 1987; Schoenian and Burfening, 1990). Ovulation rate increased with liveweight, and peak ovulation rate occurred at age 5. The mean ovulation rate was 2.26 ova (Table 2; consistent with New Zealand flocks [Davis et al., 1987; Shorten et al., 2021]) and this was associated with a mean scanning rate of 1.82 fetuses at midpregnancy. The probability of embryo survival to lambing for single ovulation was 0.96, which is consistent with direct estimates of embryo survival probability for ewes from research flocks (Shorten et al., 2013). The estimate of 0.96 was also greater than the corresponding estimates obtained from six of nine commercial flocks (Shorten et al., 2021).

**Table 2.** Table of flock statistics for mature ewes

Parameter, units	Value
Premating ewe liveweight, kg	62
Mean ovulation rate, ova	2.26
Standard deviation in ovulation rate, ova	0.53
Embryo survival probability	0.84
Lamb survival probability	0.87
Probability of conception success	0.87
Ewe age, yr	3.4
Average lamb growth rate, kg d^−1^	0.27
Average weaning weight, kg	25.8
Average number of lambs born	1.82
Average total weight of lambs weaned per ewe exposed to the ram, kg	40.4

### Between sire variability in embryo survival and ovulation rate

**Figure 3.**
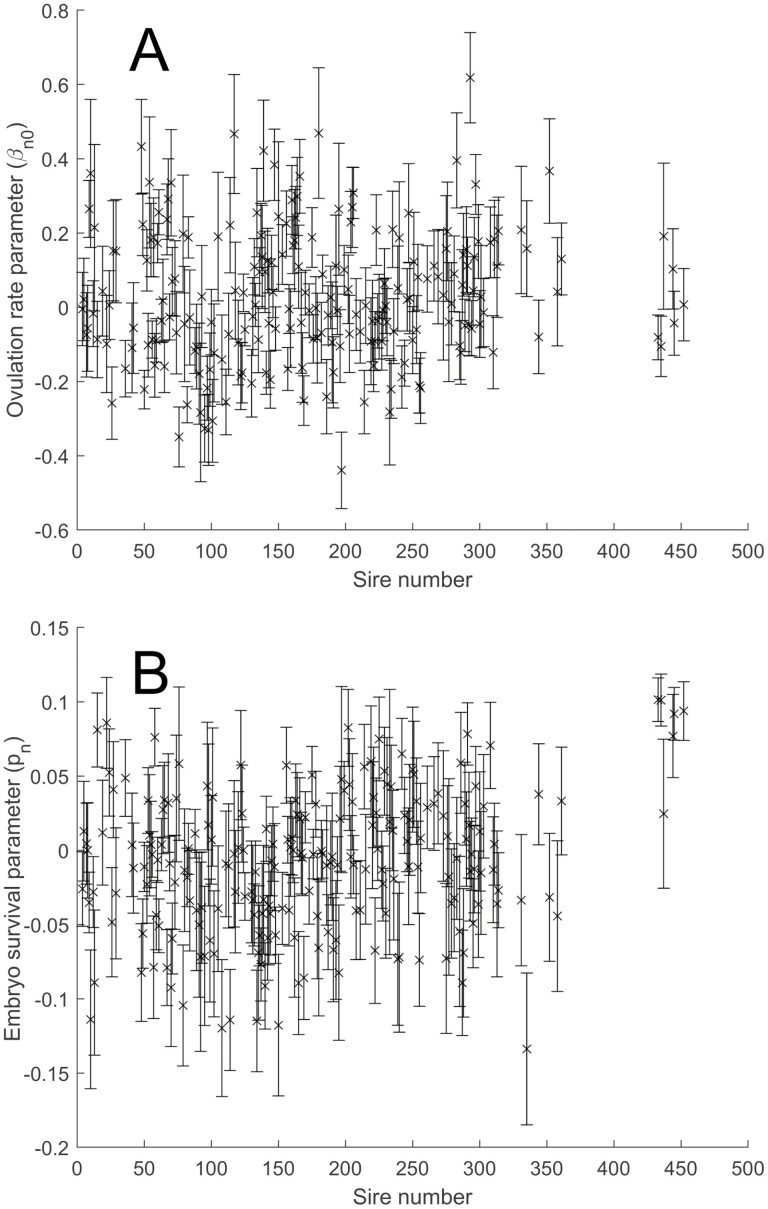
(A) Estimates of the ovulation rate parameter (β_*n*0_) for each sire (presented as a deviation from the flock average), which represents the mean number of ova for daughters of the sire without quadratic adjustment for age and premating weight (equation 1). (B) Estimates of the embryo survival parameter (*p*_*n*_) for each sire (presented as a deviation from the flock average), which is the probability that an embryo survives to lambing for daughters of the sire when there is initially a single ovum (equation 2).

Ovulation rate (β_*n*0_) and embryo survival (*p*_*n*_) model parameters (equations 1 and 2) were estimated for each sire (Fig. 3 for 208 sires). There was significant between sire variance in the estimates of ovulation rate (σ = 0.127 ova; *P* < 0.001) and embryo survival (σ = 0.026; *P* < 0.001) (Fig. 3). There was also significant between year (sire birth year) variability in the estimates of ovulation rate (σ = 0.023 ova; *P* < 0.001) and embryo survival (σ = 0.030; *P* < 0.001). The between year variability in embryo survival was larger than the between sire variability in embryo survival, highlighting the difficulty in estimating the embryo survival trait. Incorporation of other covariates and factors in the model could further reduce the between sire variability in embryo survival. These sire estimates could be used with pedigree and covariate information in a more complete genetic analysis to obtain breeding values for ovulation rate and embryo survival, although heritability estimates for embryo survival are very low (Shorten et al., 2013) and large datasets as well as careful model selection are required for embryo survival traits.

### Elasticities for the total weight of lambs weaned per ewe exposed to the ram

Model parameters for the effects of premating liveweight and age on lamb survival and lamb growth are listed in Table 1 along with flock age and premating liveweight statistics. The table of flock statistics for mature ewes is listed in Table 2. The average lamb survival probability was 0.87 and the average lamb growth rate was 0.27 kg d^−1^. The product of the average number of fetuses scanned (1.82), average lamb survival (0.87), and average weaning weight (25.8) provides an estimate of the average total weight of lambs weaned per ewe exposed to the ram (40.9 kg), although this approximation ignores, for example, covariances between these different random variables.

**Figure 4.**
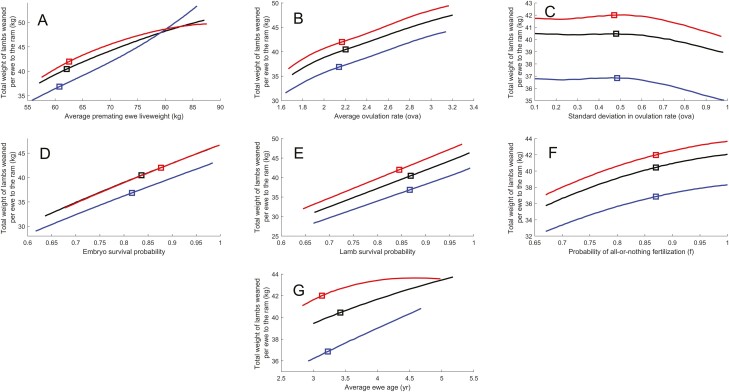
The effect of change in the (A) average premating ewe liveweight, (B) average ovulation rate, (C) standard deviation in ovulation rate, (D) embryo/fetal survival probability, (E) lamb survival probability, (F) conception success (*f*), and (G) average ewe age on the total weight of lambs weaned per ewe exposed to the ram (for mature ewes). Square symbols denote the current flock average. Lines are for all data (black), daughters of sires with high (top 50%) embryo survival (red) and daughters of sires with low (bottom 50%) embryo survival (blue). Embryo survival estimates were only obtained for sires whose daughters had more than 40 litters in total (each litter with an associated premating ewe liveweight measurement).

The effect of change in the average premating ewe liveweight, average ovulation rate, standard deviation in ovulation rate, embryo/fetal survival probability, lamb survival probability, conception success, and average ewe age on the total weight of lambs weaned per ewe exposed to the ram were largely linear and are shown in Fig. 4. The effects of perturbations in different factors on the kilograms of lamb liveweight at weaning per ewe exposed to the ram were investigated using the elasticity metric and are listed in Table 3. The elasticity metric is a unitless ratio of the percentage change in one variable to the percentage change in a second variable, when the second variable has a causal influence on the former. The elasticities describe the relative importance of the effect of average premating ewe liveweight (0.80), average ovulation rate (0.43), variability of ovulation rate (−0.01), embryo survival (0.81), lamb survival (1.01), conception success (0.36) and average ewe age (0.19) on the kilograms of lamb liveweight at weaning per ewe exposed to the ram. The largest elasticity was for lamb survival and the second largest elasticity was for embryo survival, where the elasticity for embryo survival was 80% of that for lamb survival. The elasticity for premating ewe liveweight was 0.80, indicating that a 10 kg increase in premating ewe liveweight will generate an 8 kg increase in the total weight of lambs weaned per ewe exposed to the ram. For this flock, the opportunity exists to increase flock performance through improved management of ewe premating liveweight.

**Table 3.** Table of elasticities for kilograms lambs weaned per ewe exposed to the ram for mature ewes

Parameter	Value
Premating ewe liveweight	0.80
Mean ovulation rate	0.43
Standard deviation in ovulation rate	−0.01
Embryo survival	0.81
Lamb survival	1.01
Probability of conception success	0.36
Ewe age	0.19

### Differences between the daughters of the low/high rams for embryo survival

The effect of change in the average premating ewe liveweight, average ovulation rate, standard deviation in ovulation rate, embryo/fetal survival probability, lamb survival probability, conception success, and average ewe age on the total weight of lambs weaned per ewe exposed to the ram are shown in Fig. 4 for daughters of sires with high (top 50%) and low (bottom 50%) embryo survival. Embryo survival estimates were only obtained for sires whose daughters had more than 40 litters in total (each litter with an associated premating ewe liveweight measurement), so the high/low embryo survival groups represent a subset of the flock. Embryo survival was 0.88 in the high group and 0.82 in the low group (a 6% reduction in embryo survival). These relationships were similar in shape for the high and low embryo survival groups, although the expected total weight of lambs weaned per ewe exposed to the ram was 42 kg in the high embryo survival group and 37 kg in the low embryo survival group (a 12% reduction in the total weight of lambs weaned per ewe exposed to the ram). This difference was partly mediated by differences in embryo survival and liveweight between the high/low groups, with the difference in lamb survival attributable to the lower lamb survival for the more prevalent triplet litters for the high group.

**Figure 5.**
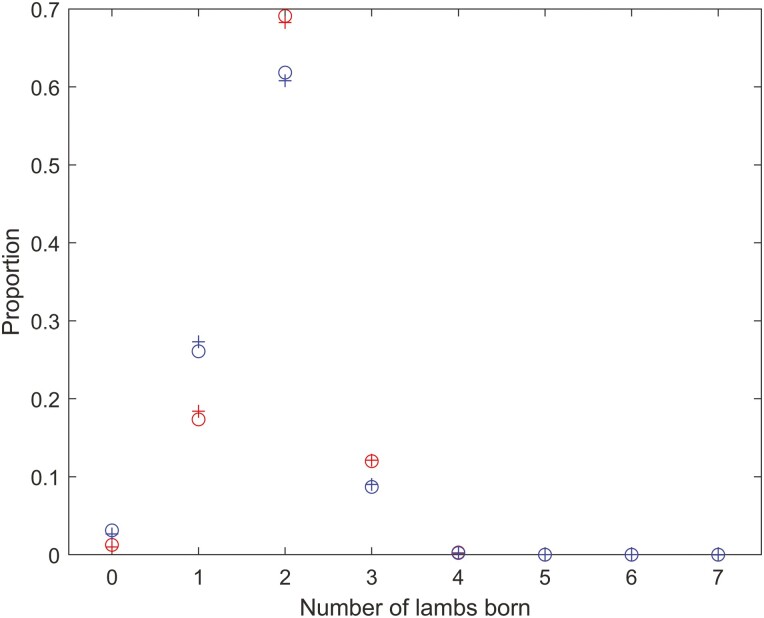
The distribution of predicted (plus symbols) and measured (circles) litter size for daughters of sires with high (top 50%) embryo survival (red) and daughters of sires with low (bottom 50%) embryo survival (blue).

**Figure 6.**
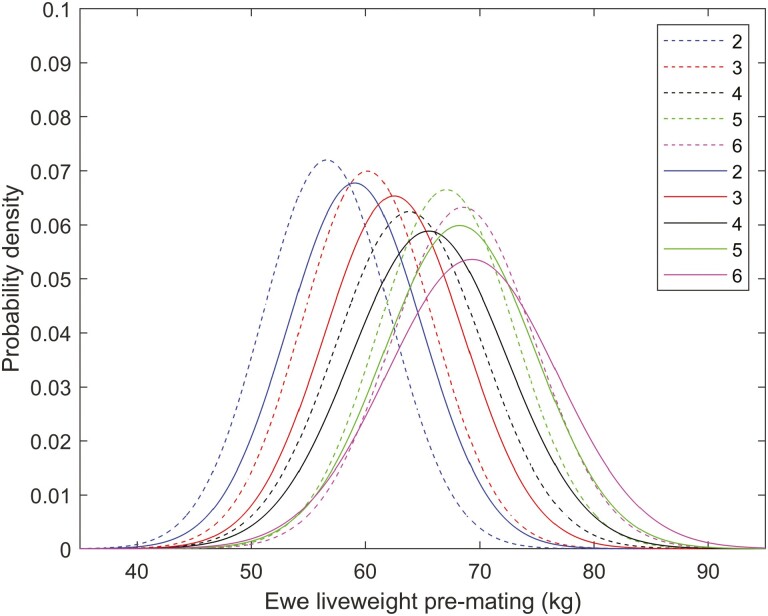
The distribution of liveweight (ewe age 2 to 6) for daughters of sires with high (top 50%) embryo survival (solid lines) and daughters of sires with low (bottom 50%) embryo survival (dashed lines).

The distribution of predicted and measured litter size for daughters of sires with high (top 50%) embryo survival and daughters of sires with low (bottom 50%) embryo survival are shown in Fig. 5. The proportion of twin litters was 70% in the high group and 60% in the low group, highlighting the potential importance of embryo survival for the rate of twinning for flocks with ovulation rates greater than two ova. The distribution of liveweight (ewe age 2 to 6) for daughters of sires with high (top 50%) embryo survival (solid lines) and daughters of sires with low (bottom 50%) embryo survival (dashed lines) are shown in Fig. 6. Liveweight was 2.5 kg lower in the low embryo survival group for age 2 to 4, with smaller differences in liveweight for age 5 to 6.

**Figure 7.**
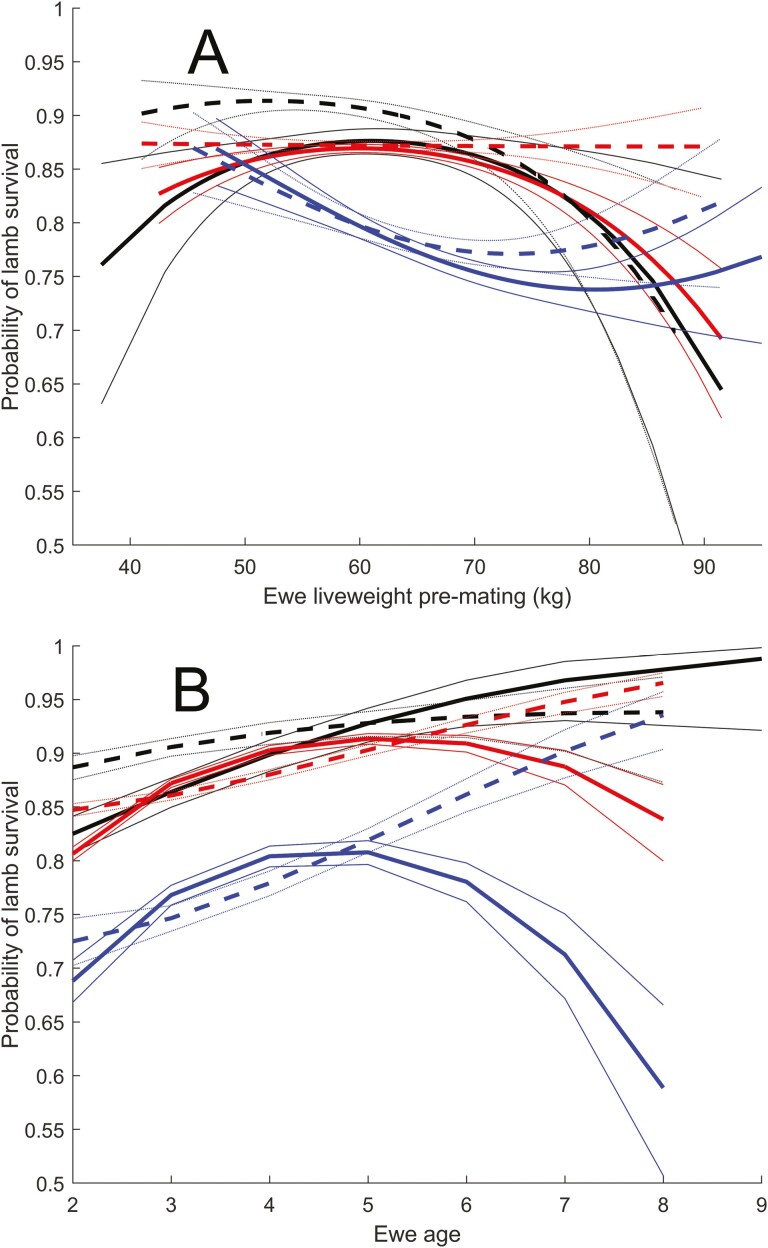
The effect of (A) liveweight and (B) age on lamb survival for litter sizes 1 (black), 2 (red), and 3 (blue) (based on average weight and age for each set of data) for daughters of sires with high (top 50%) embryo survival (solid lines) and daughters of sires with low (bottom 50%) embryo survival (dashed lines). Thin lines denote standard errors.

The effect of liveweight and age on lamb survival for litter sizes 1 to 3 (based on average weight and age for each set of data) for daughters of sires with high (top 50%) embryo survival (solid lines) and daughters of sires with low (bottom 50%) embryo survival (dashed lines) are shown in Fig. 7. There were no important functional differences in these relationships between the two groups.

**Figure 8.**
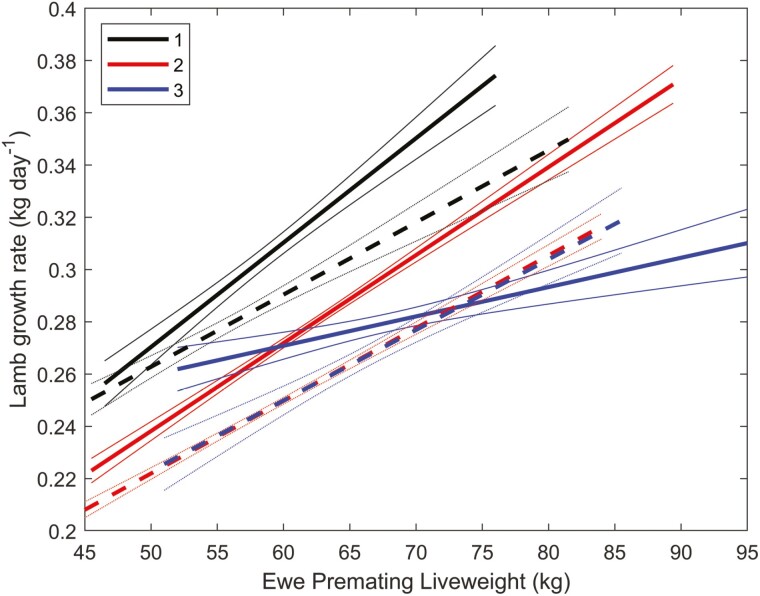
The effect of liveweight on lamb growth (litter size 1 to 3) for daughters of sires with high (top 50%) embryo survival (solid lines) and daughters of sires with low (bottom 50%) embryo survival (dotted lines). Thin lines denote standard errors.

The effect of liveweight on lamb growth (litter size 1 to 3) for daughters of sires with high (top 50%) embryo survival (solid lines) and daughters of sires with low (bottom 50%) embryo survival (dotted lines) are shown in Fig. 8. Lamb growth was reduced by 10% in the low embryo survival group for the same litter size (*P* < 0.001). The day of weaning and growth rate advantage of a ram lamb over an ewe lamb was similar for daughters of sires with high (top 50%) and low (bottom 50%) embryo survival (Table 4), highlighting that the association between embryo survival and lamb growth was not mediated by differences in these factors. This novel positive phenotypic association between embryo survival and lamb growth rate can potentially be exploited to improve flock performance and warrants further study.

**Table 4.** Table of flock statistics for daughters of sires with high (top 50%) and low (bottom 50%) embryo survival. Day of lambing and day of weaning are presented as day of year (DOY).

Parameter	High	Low
Day of lambing (all) (DOY)	271.0 ± 0.07	271.6 ± 0.07
Day of lambing (singles) (DOY)	271.8 ± 0.18	271.6 ± 0.20
Day of lambing (twins) (DOY)	271.1 ± 0.08	271.7 ± 0.09
Day of lambing (triplets) (DOY)	269.5 ± 0.22	270.7 ± 0.22
Day of lambing (quadruplets) (DOY)	268.3 ± 1.1	266.8 ± 1.3
Growth rate advantage of ram lamb over ewe lamb (kg d^−1^)	0.0266 ± 0.0028	0.0199 ± 0.0022
Day of weaning (all) (DOY)	358.7 ± 0.09	358.9 ± 0.10
Day of weaning (singles) (DOY)	360.7 ± 0.31	359.7 ± 0.30
Day of weaning (twins) (DOY)	358.8 ± 0.11	359.0 ± 0.12
Day of weaning (triplets) (DOY)	355.9 ± 0.23	356.5 ± 0.30
Day of weaning (quadruplets) (DOY)	357.1 ± 1.4	354.9 ± 1.5
